# Phytochemicals targeting Toll-like receptors 4 (TLR4) in inflammatory bowel disease

**DOI:** 10.1186/s13020-022-00611-w

**Published:** 2022-04-28

**Authors:** Wenbin Dai, Longhai Long, Xiaoqiang Wang, Sen Li, Houping Xu

**Affiliations:** 1 Geriatric Department, The Affiliated Traditional Chinese Medicine Hospital of Southwest Medical University, Luzhou, Sichuan China; 2 Spinal Surgery Department, The Affiliated Traditional Chinese Medicine Hospital of Southwest Medical University, Luzhou, Sichuan China

**Keywords:** Inflammatory bowel disease (IBD), Toll-like receptor 4 (TLR4), Phytochemicals

## Abstract

Inflammatory bowel disease (IBD) is a collective term for inflammatory diseases including Crohn’s disease and ulcerative colitis. Toll-like receptor 4 (TLR4) is thought to play a key role in the pathogenesis of IBD. Inhibition of TLR4 has been recognized as an effective target for the treatment of IBD. Many phytochemicals have been shown to have potential as new drugs for the treatment of IBD. This review surveyed the available literature and reports which focused on the in vivo effects of phytochemicals targeting TLR4 in different models of IBD, and clarified the significance of TLR4 as a current therapeutic target for IBD. Based on our review, we have concluded that phytochemicals targeting TLR4 are potentially effective candidates for developing new therapeutic drugs against IBD.

## Introduction

For thousands of years, plants have been used as the primary source of medicines [1]. Plants can produce a variety of compounds called phytochemicals [2, 3]. Phytochemicals with multiple therapeutic applications have a variety of biological functions, including anti-inflammatory, antiallergy, anti-cancer, antibiosis, anti-viral, and analgesic functions [4, 5]. Current clinical treatment of inflammatory bowel disease (IBD) is mainly based on drugs and surgery, but adverse side effects and the questionable effectiveness of drug treatment have limited their application [6, 7]. In contrast, the importance of phytochemicals in the therapeutic application of IBD have been highlighted, due to their significant efficacy and fewer side effects [8]. Phytochemicals are a good source of new anti-inflammatory drugs that can modulate various inflammatory responses and fight inflammatory diseases, especially IBD [8, 9].

IBD, including ulcerative colitis (UC) and Crohn’s disease (CD), is a group of chronic recurrent and incurable gastrointestinal diseases with an unknown etiology that can ultimately lead to the destruction of normal intestinal architecture [10]. It is an increasingly important public health issue that is receiving more and more attention. Crohn’s disease occurs primarily in the terminal ileum and adjacent colon, where inflammation can spread to the deeper layers of the intestine [11]. On the other hand, inflammation in ulcerative colitis is limited to the colonic mucosa and begins in the rectum and may involve the entire colon [12]. IBD is known to be caused by an inappropriate response of a dysfunctional mucosal immune system to resident microbiota and other harmful antigens [13]. Additionally, dysfunction of the immune system, particularly Toll-like receptor 4 (TLR4) dysfunction, plays a key role in the pathogenesis of IBD [14]. Although many drugs have been developed to treat IBD, these drugs have adverse side effects on the gastrointestinal tract [15]. Therefore, targeting TLR4 is considered a new therapeutic strategy for patients with IBD [16]. Numerous studies have shown that phytochemicals, including phenolic compounds, terpenoids, alkaloids, and organosulfur compounds, can act as therapeutic agents and exert protective and therapeutic effects on IBD [17]. In particular, some phytochemicals are being studied as antagonists of TLR4 [18]. Considering the advantages of phytochemicals, and their fewer side effects, phytochemicals targeting TLR4 represent a potentially good source of new drugs for the treatment of IBD [14]. The study of the role of phytochemicals targeting TLR4 in IBD contributes to providing a firm theoretical basis for the development of drugs for the treatment of IBD. In the process of researching articles, we found that most studies to date have been based on animal models and cell experiments. Thus, further extensive clinical studies focusing on the efficacy of phytochemicals against IBD via TLR4 should be conducted.

For this review, we searched for relevant articles published between 2011 and December 2021 in PubMed/Medline, using different combinations of key terms including “TLR4”, “inflammatory bowel disease”, “IBD”, “colitis”, “intestinal inflammation”, and “phytochemical”. Articles in our search had to meet the following criteria: (a) the focus was on a natural phytochemical that affects IBD via TLR4, (b) pharmacological studies in vivo animal models, and (c) only English language articles; all papers that did not meet these criteria were excluded. It is important to note that some relevant articles may not have been included in this study because the search strategy we employed was not absolutely perfect.

## Toll-like receptor

As the most important pathogen pattern recognition molecules (PPRs), Toll-like receptors (TLRs) are responsible for the activation and association of innate and adaptive immune responses and play a key role in maintaining homeostasis in the gut.

TLRs detect a wide range of pathogen-associated molecular patterns (PAMPs), including Gram-negative and positive bacteria, viruses, nucleic acids, flagellin proteins, lipids, and damage-associated molecular patterns (DAMPs) [19, 20]. TLRs is a family of transmembrane receptors and 13 TLRs have been identified in humans [21]. TLRs contain extracellular leucine-rich repeat (LRR) motifs and cytoplasmic Toll/interleukin-1 receptor (TIR) homology domain. The TIR domain mediates interactions between TLRs and TIR-domain-containing adaptor proteins, leading to the biological specificity of the TLR response [22, 23]. Mammalian TLRs induce a variety of effector molecules, such as iNOS and antimicrobial peptides that can directly destroy microbial pathogens [24]. TLRs are well expressed in different cells or tissues such as dendritic cells, natural killing, epithelium cells, and macrophages [25]. When TLRs are activated, together with the contribution of the secreted proteins myeloid differentiation factor 2 (MD-2) and cluster of differentiation 14 (CD14), they stimulate downstream signal transduction procedures, such as inducing a variety of inflammatory cytokines through transcription by mediating the phosphorylation of IκB to activate NF-κB [13, 26]. This leads to the secretion of pro-inflammatory mediators, which directly affect the immune response [27]. Additionally, the expression of innate immune receptors plays a key role in the abnormal and enhanced inflammatory response. As a result, the mutation and dysregulation of TLRs will lead to the over-generation of IBD [28].

The association between dysregulation of the innate immune system and the emergence of several diseases, such as inflammation, autoimmunity, and cancer has made it the focus of the development of many agents countering uncontrolled TLR-mediated signaling, as these receptors are primary regulators of the host’s innate immunity [29]. TLR antagonists have been mainly explored as structural analogs of agonists, which block agonistic action of TLR ligands through binding to the receptor and preventing the propagation of the downstream inflammatory/autoimmune cascades [30, 31]. TLR antagonists are currently in clinical trials for the treatment of septic shock and autoimmune diseases [32]. New evidence suggests that TLR agonists are a promising class of immunomodulatory agents that provide long-term protection against subsequent infectious challenges by enhanced innate immunity [33, 34]. TLR agonists induce augmentation of cell recruitment, antimicrobial effector functions (i.e. respiratory burst, phagocytosis, production of proinflammatory cytokines and chemokines), attenuate inflammation, bacterial clearance, and trigger cross-protection to infection with clinically related pathogens [35]. A number of TLR agonist and antagonist compounds have been designed recently which are able to target specific innate immune receptors (Table 1).

**Table 1:** Therapeutic applications of TLR agonists and antagonists

Class	Drug	Explanation	References
TLR2 agonist	SMP-105	It has been approved for the treatment of bladder cancer	[36]
TLR3 agonist	Poly-IC12U	It is used in combination with vaccines for the treatment of ovarian, breast, colorectal, and brain tumors	[37]
TLR4 antagonist	TAK-242	It could enhance the sensitivity of cancer cells to doxorubicin and cisplatin, respectively	[38]
TLR4 agonist	MPLA	MPLA (monophosphoryl lipid A) is the only TLR4 agonist to be approved by the FDA for the use as a vaccine adjuvant in humans (Cervarix^®^, Fendrix^®^)	[39, 40]
TLR2/4 agonist	BCG	BCG (bacillus Calmette–Guerin) has been approved by the FDA for intravesical treatment of bladder carcinoma in situ and superficial bladder cancers	[41]
TLR7 agonist	Imiquimod	Imiquimod is an FDA approved immune response modifier for the topical treatment of genital warts caused by HPV and has also been proposed as a therapeutic adjunct for COVID-19 and related infections	[42, 43]
TLR9 antagonist	IROs	IROs (immunoregulatory oligonucleotides) can be used to suppress autoimmune and inflammatory diseases	[44]

## The role of TLR4 in IBD

**Fig. 1:**
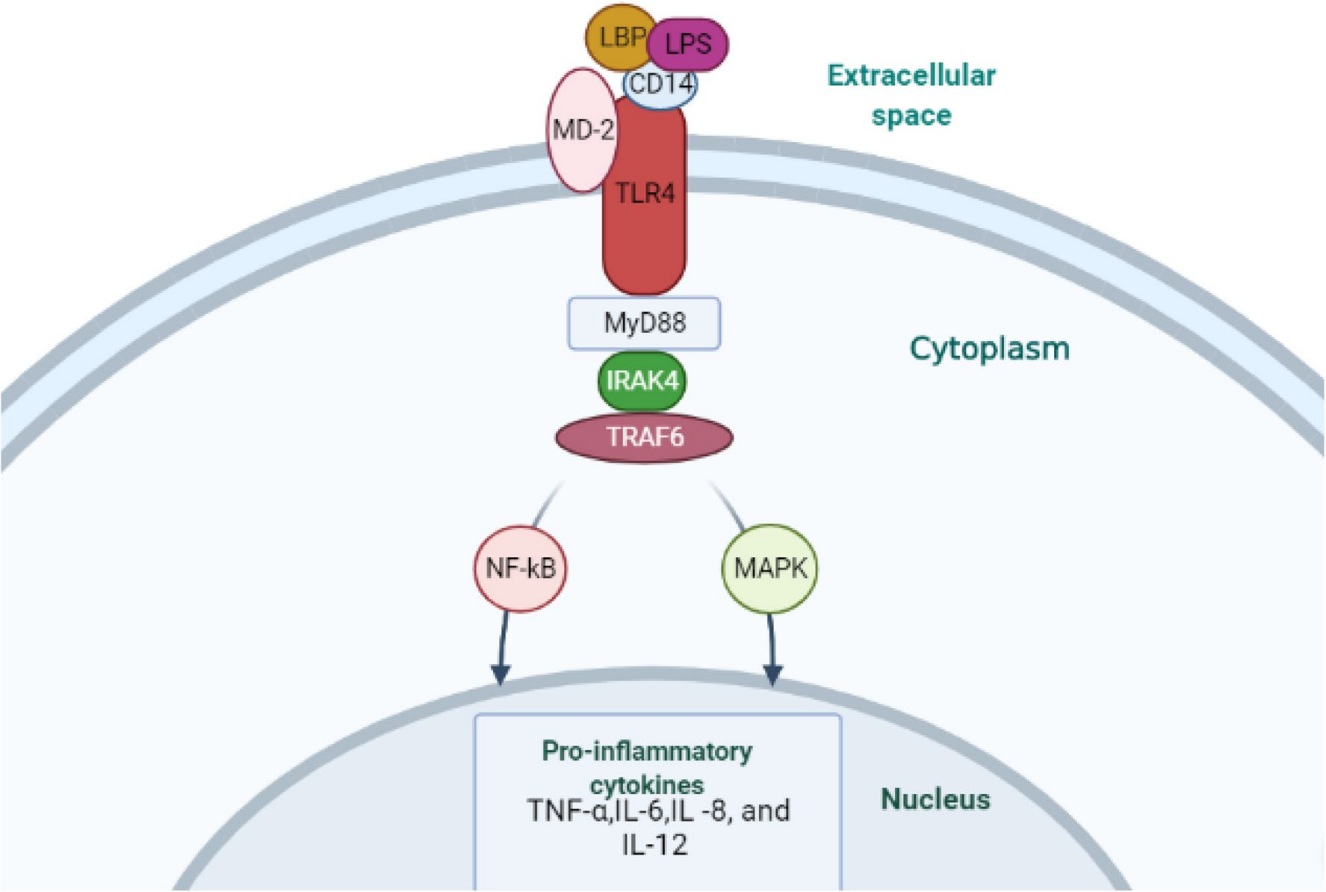
Toll-like receptor (TLR) 4 signaling pathway

TLR4, widely expressed in various immune cells and epithelial/endothelial cells, is a key member of TLR family and a classical mediator of inflammation and acts as a signaling molecule between innate and adaptive immunity, as well as between inflammation and infection [45, 46]. TLR4 has been identified as the primary pattern recognition receptor (PRR) as well as the canonical receptor for Gram-negative bacteria’s lipopolysaccharide (LPS), and there is emerging evidence that supports that TLR4 is involved in homeostasis, apoptosis, intestinal inflammation, and inflammatory bowel disease [47–49]. TLR4 combined with LPS binding protein (LBP), CD14 and MD-2, acts as PRR for LPS of gram-negative bacteria (Fig. 1) [28]. When LPS is identified, LBP will transfer LPS to cell surface CD14 and then bind to the TLR4/MD-2 receptor complex [50]. Subsequently, TLR4 is activated by the formed LPS–LBP–CD14 complex, signaling through adaptor protein myeloid differentiation 88 (MyD88) and serine kinase IL-1R-associated kinase 4 (IRAK4) and the adaptor protein TNF receptor-associated factor 6 (TRAF6). Then, NF-κB and mitogen-activated protein kinase (MAPK) are activated by this pathway, leading to the transcription of proinflammatory cytokines, such as tumor necrosis factor (TNF)-α and IL-6, -8, and -12, and the initiation of IBD [51–53].

Under normal conditions, normal or low expression of TLR4 in intestine controls inflammation, but elevated TLR4 expression in the intestinal mucosa of patients with IBD leads to sustained secretion of inflammatory cytokines and ultimately to the development of intestinal inflammation [54, 55]. Therefore, the occurrence and development of IBD may be related to the abnormal expression of TLR4 in intestinal epithelium. Significantly increased mRNA and protein expression of TLR4 was reported in the colonic mucosa of patients with UC and CD compared to healthy controls [56, 57], similarly suggesting that TLR4 is involved in IBD. Therefore, targeting and inhibiting TLR4 is effective for the treatment of IBD [16].

## Phytochemicals targeting the TLR4

Current treatments for IBD can have many side effects, such as fatigue, nausea, abdominal pain, and diarrhoea [15]. Surprisingly, dietary supplements of plant-derived natural compounds are considered to have therapeutic protective and therapeutic effects for IBD [17]. Here, we discuss the present evidence that phytochemicals could induce IBD remission by affecting the TLR4 in animal model systems of IBD (Table 2).

**Table 2:** Phytochemicals targeting TLR4 in inflammatory bowel disease models

Class of phytochemicals	Phytochemical name	Main source	Study model	Dosage	References
Phenolic compounds	Curcumin	Turmeric (*Curcumin longa*)	TNBS-induced	100 mg/kg (oral)	[58]
	Baicalin	*Radix Scutellar-ae*	TNBS-induced	1.25, 2.5, 5 mg/ml (oral)	[59]
	Naringenin	Grapefruit	DSS-induced	50 mg/kg (oral)	[60]
	Eriodictyol	Yerba Santa Clause *(Eriodictyon californicum)*	TNBS-induced	5, 20, 50 mg/kg (oral)	[61]
Alkaloids	Oxyberberine	*Coptidis chinensis Franch*	DSS-induced	12.5, 25, 50 mg/kg (oral)	[62]
	Dihydroberberine	*Coptis chinensis Franch*	DSS-induced	12.5, 25, 50 mg/kg (oral)	[63]
	Piperine	Black pepper *(Piper nigrum)* and long pepper *(Piper longum)*	FFA-induced	5, 10 mg/kg (oral)	[64]
Terpenoids	Miltirone	*Salvia miltiorrhiza Bunge*	TNBS-induced	5, 15, 45 mg/kg (oral)	[65]
	Paeoniflorin	*Paeonia lactiflora Pall*	DSS-induced	50 mg/kg (oral)	[66]

### Phenolic compounds

Phenolics is a group of secondary plant metabolites with several classes, including phenolic acids, flavonoids, stilbenes, coumarins, lignins, and tannins [67]. Numerous studies have shown that polyphenols are effective in treating IBD [68, 69].

#### Curcumin

Curcumin, a plant-derived polyphenolic compound, is naturally present in turmeric (*Curcuma longa*) and is known to exhibit a variety of pharmacological effects including anti-inflammatory, and anti-tumorigenic [70]. Accumulating evidence has revealed that curcumin has anti-inflammatory effects against IBD. A study reported that curcumin improves 2,4,6-trinitrobenzene sulfonic acid (TNBS)-induced colitis in rats [71]. To investigate the upstream signaling mechanism of curcumin in experimental colitis in rats, Zhaojing Zeng et al. used experimental colitis induced in rats by intra-rectal instillation of TNBS. They found that the levels of TLR4 and NF-κB were significantly upregulated in the inflammatory colon. In contrast with the model group, the curcumin remarkably lowered the levels of TLR4 and NF-κB. In addition, curcumin treatment inhibited Myeloperoxidase (MPO) activity. Curcumin targeting TLR4 may be considered as a putative candidate of treatment in IBD [58].

#### Baicalin

Baicalin is a kind of flavonoid derived from the roots of traditional Chinese medicine *Scutellariae Radix* [72], which shows a variety of biological activities such as diminishing inflammation, reducing cellular lipid anabolism, as well as inhibiting bacterial and viral infection [73, 74]. It has been reported that baicalin reduced serum levels of proinflammatory factors IL-1β, IL-6 and CRP [75]. Baicalin ameliorated TNBS-induced colitis injury by suppressing TLR4 signaling in a concentration-dependent manner, inhibiting NF-κB activation and limiting the inflammatory response, such as ICAM-1, MCP-1, Cox-2, TNF-α, IL-1β and IL-6 [59].

#### Naringenin

Naringenin is a citrus flavonoid mainly derived from grapefruit, which has been reported to have antioxidant, anti-inflammatory properties [76, 77]. A recent report showed that the oxidative damage and injury of colon tissues in a mice model of acetic acid-induced colitis was ameliorated by naringenin [78]. It is also reported that naringenin significantly improved colitis in a DSS-induced mice colitis model by inhibiting of TLR4 protein and NF-κB activity, downregulating the expression of inflammatory mediators (iNOS, ICAM-1, MCP-1 Cox-2, TNF-α and IL-6) and the production of inflammatory cytokines (TNF-α and IL-6) [60].

#### Eriodictyol

Eriodictyol is a natural flavanone mainly isolated from yerba Santa Clause (*Eriodictyon californicum*), a plant local to North America, which has various physiological functions, including anti-inflammation, anti-oxidation, analgesic effects, neuroprotective effects, improving diabetes and diabetic complications [79]. It has been reported to alleviate cisplatin-induced kidney injury by inhibiting inflammation and oxidative stress [80]. Recently, a study demonstrated that eriodictyol alleviated TNBS-induced intestinal tissue injury in rats through repressing TLR4/NF-κB signaling pathway and reducing pro-inflammatory cytokines levels, such as TNF-α, IL-6, IL-1β, IL-2, and IL-12 [61].

### Alkaloids

Alkaloids is a large group of chemicals characterized by the presence of nitrogen in their structure, which could not only downregulate inflammatory cytokines production, reduce oxidative stress, but also inhibit NF-κB levels [81]. An alkaloid was reported to improve the intestinal structure and barrier function in a DSS-induced colitis model [82].

#### Oxyberberine

Oxyberberine is an oxidized protoberberine alkaloid isolated from the *Coptidis chinensis Franch* [62]. It has been revealed that oxyberberine has a lot of pharmacological actions including anti-inflammatory, anti-tumor and anti-arrhythmic [83–85]. A research reported that oxyberberine alleviated intestinal mucosal inflammation and colonic mucosal injury in DSS-induced colitis mice by suppressing the TLR4–MyD88–NF-κB signaling pathway [62].

#### Dihydroberberine

Dihydroberberine is a natural occurring isoquinoline alkaloid extracted from the *Coptidis chinensis Franch*, which is a hydrogenated derivative of berberine [86]. Furthermore, numerous studies have shown that it has stronger anti-inflammatory, anti-atherosclerotic and hypolipidemic activities than berberine does [87]. It was shown that dihydroberberine treatment observably blocked the TLR4/MyD88/NF-κB signaling pathway by inhibiting the protein expression of TLR4, MyD88 and p-IκBα, which in turn suppressed the inflammatory responses and restored gut barrier function. Therefore, dihydroberberine produced a significant protective effect on DSS-induced colitis models [63].

#### Piperine

Piper species have multiple effects and have been used in traditional medicine to treat a variety of diseases, such as menstrual pain, sleeping problems, tuberculosis, chronic gut-related pain, respiratory tract infections, and arthritic conditions [88].

Piperine was the primary lipophilic component extracted from black pepper (*Piper nigrum*) and long pepper (*Piper longum*), which has been reported to be effective against metabolic syndrome and have anti-inflammatory activity [89]. Piperine significantly reduced inflammatory mediators in DSS-induced colitis [90]. According to one research, piperidine not only inhibited the abnormal secretion of pro-inflammatory mediators, including NO, cytokine TNF-α, but also reduced free fatty acid (FFA)-induced TLR4-mediated inflammation [64].

### Terpenoids

Terpenoids is a large group of chemicals produced mainly by flowering plants, and to date, about one thousand terpenoids have been isolated [91]. According to the number of their isoprene units, they are divided into several categories, including hemiterpenoids, monoterpenoids, sesquiterpenoids, etc. [92]. It has been shown that terpenoids ameliorated experimental colitis by reducing colonic injury and inflammation and possibly by decreasing permeability [93].

#### Miltirone

Miltirone is one of the bioactive diterpene quinones extracted from *Salvia miltiorrhiza Bunge*. It has been reported to have a wide range of activities such as antioxidant, anti-inflammatory effects, etc. [94, 95]. Miltirone could significantly ameliorate the clinical symptoms of TNBS-induced IBD in mice by reducing the levels of inflammatory cytokines, decreasing the protein and mRNA level of IQGAP2, TLR4, MyD88, NF-kB p65. Therefore, the anti-inflammation effect of miltirone in IBD may be related to the TLR4/NF-kB/IQGAP2 signaling pathway [65].

#### Paeoniflorin

Paeoniflorin is the main bioactive component of *Paeonia lactiflora Pall*, which is a water-soluble monoterpene glycoside [96]. Paeonia root is one of the famous natural medicines in China, which has been used as medicine in traditional Chinese medicine for thousands of years [97]. Paeoniflorin has been reported to have anti-inflammatory, immunomodulatory, and anti-arthritic effects [98, 99]. A recent investigation (article in Chinese) indicated that the symptoms of oxazolone-induced colitis was effectively improved by paeoniflorin [100]. Paeoniflorin treatment was reported to lead to significant improvement of DSS-induced colitis by significantly reversing the upregulation of TLR4, decreasing the activity of MPO, reducing the production of inflammatory cytokines (TNF-αand IL-6), downregulating inflammatory mediators (MCP-1, Cox-2, IFN-γ, TNF-α, IL-6, and IL-17), and limiting the inflammatory (histological) response [66].

## Conclusions and perspective

Today, the incidence of inflammatory bowel disease is increasing in Asian countries, including China, with globalization and lifestyle changes, especially dietary habits. Previous studies have identified that TLR4 signaling pathway plays a key role in the development of IBD. Therefore, targeting the TLR4 signaling pathway for the treatment of IBD is an effective approach. The nucleotide-binding domain and leucine-rich repeat containing (NLR) family is also an important factor to release cytokines and to form the inflammasome, which can form multiprotein complexes termed “inflammasomes” [101]. Among these inflammasomes, the NLRP3 inflammasome, one of NLRs, has been extensively studied of late [102]. Recent studies have suggested that NLRP3 governed the productions of pro-inflammatory cytokines, and is associated with the pathogenesis of more common inflammatory diseases [103]. TLR4 has been reported to regulate the activation of NF-κB p65, which affects the expression of NLRP3/IL-1β [104]. Therefore, targeting TLR4 synergistically with NLRP3 is also an effective way to treat IBD. There are many synthetic drugs being used to treat IBD, but the problem with these drugs is that they have many side effects. However, phytochemicals have been reported to have significant advantages in the treatment of IBD with few negative outcomes. Therefore, it is prudent to consider phytochemicals that modulate TLR4 signaling pathway as potential candidates for IBD treatment, but there are still challenges in bioavailability and delivery. Specifically, this review has focused on phytochemicals that inhibit TLR4 signaling pathway and ameliorate IBD symptoms in vivo in animal model systems. Unfortunately, to date, few clinical trials have been conducted targeting IBD pathobiology by TLR4 modulators. This raises the importance of further experiments and clinical trials focusing on IBD therapy through TLR4-modulating agents. In the future, numerous clinical studies are needed to validate the beneficial effects of phytochemicals targeting TLR4 in the treatment of IBD and to develop new drugs. In the current context of global environmental change, and with biodiversity declining significantly, conserving medicinal plants for treating human illness and maintaining the integrity of the associated local ecological knowledge were recognized as an important component of the Sustainable Development Goal [105]. However, the decline in plant and animal species and the global reduction in the area of nature reserves are challenges affecting biodiversity. Therefore, increasing international cooperation is needed to protect biodiversity.

## Data Availability

All data are available in the manuscript and they are showed in figures and tables.

## Abbreviations

BCG: Bacillus Calmette–Guerin
CD: Crohn’s disease
CD14: Cluster of differentiation 14
COVID-19: Corona Virus Disease 2019
Cox-2: Cyclo-oxygenase-2
CRP: C-reaction protein
DAMPs: Damage-associated molecular patterns
DSS: Dextran sulphate sodium
FDA: U.S. Food and Drug Administration
FFA: Free fatty acid
HPV: Human papillomavirus
IBD: Inflammatory bowel disease
ICAM-1: Intercellular adhesion molecule-1
IFN-γ: Interferon-γ
IL: Interleukin
iNOS: Inducible nitric oxide synthase
IQGAP2: IQ-domain GTPase-activating protein 2
IRAK4: IL-1R-associated kinase 4
IROs: Immunoregulatory oligonucleotides
IκB: IkappaB
LPS: Lipopolysaccharide
LRR: Leucine-rich repeat
MAPK: Mitogen-activated protein kinase
MCP-1: Monocyte chemoattractant protein-1
MD-2: Myeloid differentiation factor 2
MPLA: Monophosphoryl lipid A
MPO: Myeloperoxidase
mRNA: Messenger RNA
MyD88: Myeloid differentiation 88
NF-κB: Nuclear factor-kappaB
NLRs: NOD-like receptors
NO: Nitric oxide
PAMPs: Pathogen-associated molecular patterns
PPRs: Pathogen pattern recognition molecules
PRR: Primary pattern recognition receptor
TIR: Toll/interleukin-1 receptor
TLR4: Toll-like receptor 4
TLRs: Toll-like receptors
TNBS: 2,4,6-Trinitrobenzene sulfonic acid
TNF-α: Tumor necrosis factor-α
TRAF6: TNF receptor-associated factor 6
UC: Ulcerative colitis
